# Hb Titusville异常血红蛋白病1例

**DOI:** 10.3760/cma.j.issn.0253-2727.2023.12.017

**Published:** 2023-12

**Authors:** 艳芬 郑, 丽娟 张

**Affiliations:** 中山大学附属第五医院临床检验科，珠海 519000 Department of Clinical Laboratory, the Fifth Affiliated Hospital of Sun Yat-sen University, Zhuhai 519000, China

患者，男，37岁。患者诉1年前无明显诱因出现阵发性头晕恶心，行走不利，胸闷，无气促、呼吸困难。1个月前完善颅脑影像学检查，排除脑出血、脑梗死及占位性病变的可能性，诊断为前庭性眩晕，予对症处理后症状仍反复出现。患者再次就诊，血气分析示动脉血氧分压（PaO_2_）为113 mmHg（1 mmHg＝0.133 kPa），血氧饱和度（SaO_2_）为87.6％。患者初步诊断考虑低氧血症。结合患者有长期吸烟史，不除外由慢性阻塞性肺病、支气管哮喘导致，收入我院呼吸科住院治疗。

入院后予低流量给氧、止晕等对症处理，患者头晕症状未明显改善，无气促、呼吸困难，监测指尖SaO_2_仍偏低，波动在88％～93％。患者入院时血气分析结果示SaO_2_偏低而PaO_2_正常，趋势不一致，不排除采样混入空气导致。复查血气分析示PaO_2_正常或偏高、SaO_2_偏低，两次检测结果分别为PaO_2_ 110 mmHg、SaO_2_ 87.90％及PaO_2_ 103 mmHg、SaO_2_ 86.20％。因此可排除采样误差导致二者趋势不一致。此外，肺功能、胸部CT结果未提示明显的呼吸系统疾病导致患者的低氧血症。心脏超声、动态心电图结果亦未提示循环系统疾病，其余颈椎CT、颅脑磁共振成像未提示明显异常。综合上述结果，暂不考虑呼吸系统、循环系统疾病导致的低氧血症。血常规示HGB 155 g/L，平均红细胞体积（MCV）91.20 fl，红细胞平均血红蛋白含量（MCH）30.90 pg，红细胞平均血红蛋白浓度（MCHC）339 g/L。患者血常规虽未提示明显异常，但未能排除该患者是否存在异常血红蛋白，由于血红蛋白的结构异常可导致携氧能力下降，进而导致低氧血症，而PaO_2_则正常，目前已有报道的血红蛋白异构体如Hb Titusville、Hb Bassett携带者可表现为低SaO_2_而PaO_2_正常。结合患者入院后在吸氧条件下，SaO_2_改善不明显，而PaO_2_正常，这一现象也提示异常血红蛋白存在的可能。高效液相色谱法行血红蛋白电泳结果示：HbF or Hb variant 16.4％，HbA2 2.1％，HbA 81.0％。为检测珠蛋白是否存在突变，进一步对珠蛋白进行DNA测序分析，发现患者α1珠蛋白链第94号密码子存在突变（GAC>AAC），编码氨基酸由天冬氨酸（Asp）变为天冬酰胺（Asn）（图1），突变对应的血红蛋白异构体为Hb Titusville，该异构体特点为低氧亲和性。因此，患者低氧血症考虑与其为Hb Titusville携带者相关，有文献报道Hb Titusville携带者并无明显临床症状，主要表现为SaO_2_偏低而PaO_2_正常，考虑患者头晕症状与Hb Titusville导致的低氧血症无关。对该变异体的识别使患者免于广泛的、不必要的医学检查。目前全世界报告的Hb Titusville病例约有20例，来自不同的背景，如高加索、西班牙、以色列、印度和北欧。本研究为中国报告的首例Hb Titusville。

**图1:**
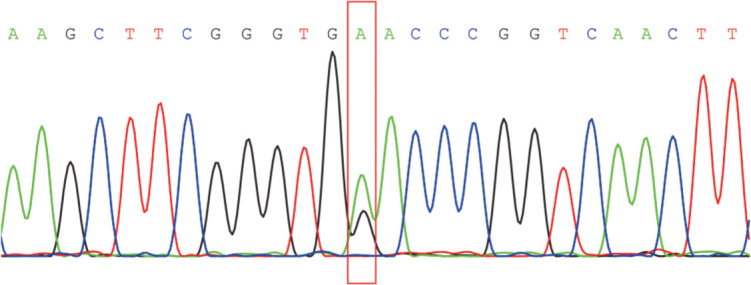
Hb Titusville患者血红蛋白α1珠蛋白链Sanger测序示c.94 GAC>AAC (p.Asp94Asn)

